# PGC-1α-Coordinated Hypothalamic Antioxidant Defense Is Linked to SP1-LanCL1 Axis during High-Fat-Diet-Induced Obesity in Male Mice

**DOI:** 10.3390/antiox13020252

**Published:** 2024-02-19

**Authors:** Shuai Shi, Jichen Wang, Huan Gong, Xiaohua Huang, Bin Mu, Xiangyu Cheng, Bin Feng, Lanlan Jia, Qihui Luo, Wentao Liu, Zhengli Chen, Chao Huang

**Affiliations:** 1 Laboratory of Experimental Animal Disease Model, College of Veterinary Medicine, Sichuan Agricultural University, Chengdu 611130, China; shishuaikeco@163.com (S.S.); saujacinthwang@163.com (J.W.); gh_3107@163.com (H.G.); mb616@163.com (B.M.); xiangyu16139@163.com (X.C.); jialanlan@sicau.edu.cn (L.J.); lqhbiology@163.com (Q.L.); liuwt1986@126.com (W.L.); 2 Key Laboratory of Animal Disease and Human Health of Sichuan Province, College of Veterinary Medicine, Sichuan Agricultural University, Chengdu 611130, China; 3 Animal Nutrition Institute, Sichuan Agricultural University, Chengdu 611130, China; hxh3028@163.com (X.H.); fengbin@sicau.edu.cn (B.F.)

**Keywords:** obesity, oxidative stress, hypothalamus, inflammation, LanCL1

## Abstract

High-fat-diet (HFD)-induced obesity parallels hypothalamic inflammation and oxidative stress, but the correlations between them are not well-defined. Here, with mouse models targeting the antioxidant gene *LanCL1* in the hypothalamus, we demonstrate that impaired hypothalamic antioxidant defense aggravates HFD-induced hypothalamic inflammation and obesity progress, and these could be improved in mice with elevated hypothalamic antioxidant defense. We also show that peroxisome proliferator-activated receptor γ coactivator 1α (PGC-1α), a critical transcriptional coactivator, is implicated in regulating hypothalamic LanCL1 transcription, in collaboration with SP1 through a direct interaction, in response to HFD-induced palmitic acid (PA) accumulation. According to our results, when exposed to HFD, mice undergo a process of overwhelming hypothalamic antioxidant defense; short-time HFD exposure induces ROS production to activate PGC-1α and elevate LanCL1-mediated antioxidant defense, while long-time exposure promotes ubiquitin-mediated PGC-1α degradation and suppresses LanCL1 expression. Our findings show the critical importance of the hypothalamic PGC-1α-SP1-LanCL1 axis in regulating HFD-induced obesity, and provide new insights describing the correlations of hypothalamic inflammation and oxidative stress during this process.

## 1. Introduction

Obesity has become a global epidemic with a fast-growing prevalence. Defined as an imbalanced homeostasis of body fat mass, obesity is caused by both genetic and environmental factors [1], affecting more than one billion adults in the world [2]. Obesity increases the risk of numerous complications, such as diabetes, hypertension, and cardiovascular diseases, which are all serious threats to human health [3]. Chronic and low-grade inflammation induced by obesity could affect most human organs, and injuries to some of these organs are in turn implicated in the development and progress of obesity with complex correlations [4]. Over recent decades, accumulating findings have revealed the intricate functions of the hypothalamus, a brain area that integrates metabolic feedback and regulates energy homeostasis, in regulating the pathogenesis of obesity [5,6,7,8]. On the one hand, the factors that commonly promote obesity development can induce hypothalamic dysfunction or structural damages; on the other hand, hypothalamic injuries have been linked to the development and progress of obesity [7,8,9,10]. Some cellular biological processes and toxic reactions in the hypothalamus contribute to the correlations between hypothalamic function and obesity progress, especially neuronal inflammation and oxidative stress. Hypothalamic inflammation is present as an early event in obesity development [11], and the interference of inflammatory pathways suppresses the progress of obesity [12,13,14]. Similarly, oxidative stress and damage are present in the hypothalamus of obesity models [15,16] and hypothalamic oxidative stress aggravates obesity-induced insulin and leptin resistance [17]. However, the correlation between hypothalamic inflammation and oxidative stress, as well as how this correlation affects the development and progress of obesity, is not well-defined.

Lanthionine synthase C (LanC) is a protease that catalyzes the crosslinking of sulfide groups in polypeptide chains to participate in the synthesis of antimicrobial peptides in prokaryotes [18]. LanC has three mammalian homologs (LanC-like proteins, LanCLs): LanCL1, LanCL2, and LanCL3. Among them, *LanCL3* is considered to be a pseudogene [19]. Previously, we first reported the biological function of LanCL1 in mammals. As a GSH-binding protein highly expressed in brain neurons and testes, LanCL1 has GSH-dependent antioxidant activity and is essential for the redox homeostasis of developing neurons [20]. Also, overexpression of LanCL1 could significantly delay the onset of ALS in mice, improve their motor dysfunction, and prolong their lifespan [21] through its antioxidant activity. Moreover, we also found that SP1 activity is required for ROS-induced LanCL1 expression, providing evidence linking the SP1–LanCL1 axis to cellular antioxidant defense [22]. The antioxidant activity of LanCL1 was also reported in multiple in vitro studies [23,24,25,26]. In this study, using mouse models in which LanCL1 is specifically knocked out or knocked in in hypothalamic neurons, we aim to evaluate the impacts of altered hypothalamic antioxidant defense in obesity progress, as well as the correlations between hypothalamic oxidative stress and inflammation induced by obesity. We found that neuronal-expressed LanCL1 in the hypothalamus was highly correlated with the progress of HFD-induced obesity. A deficiency of hypothalamic LanCL1 aggravated obesity-induced metabolic dysfunctions and hypothalamic inflammation, while an overexpression of hypothalamic LanCL1 was protective against these defects. Finally, we noticed that the important metabolic regulator PGC-1α, through its interaction with SP1, was involved in regulating the SP1–LanCL1 axis to cope with hypothalamic oxidative stress caused by obesity.

## 2. Materials and Methods

### 2.1. Generation and Validation of Genetically Modified LanCL1 Mice

The LanCL1 floxed mice and Rosa26-LanCL1-V5 transgenic mice were designed and obtained as previously described [20,22], and the rip-Cre transgenic mice were obtained from the Jackson Laboratory (Bar Harbor, ME, USA). A series of mating processes with homozygous LanCL1 floxed mice (LanCL1^f/f^) and rip-Cre mice were performed to obtain LanCL1 cKO mice. Also, Rosa26-LanCL1-V5 transgenic mice were crossed with rip-Cre transgenic mice to obtain LanCL1 cKI mice. All of these mice were genotyped with PCR using the primers shown in Table 1. The protein expression of hypothalamic LanCL1 in cKO and cKI mice was validated with Western blots using an affinity-purified rabbit polyclonal LanCL1 antibody we generated before [22]. All mouse experiments conducted in this study were performed in accordance with the Animal Care and Use Committee guidelines of Sichuan Agricultural University. The mice were housed under specific-pathogen-free (SPF) conditions in a standard individual ventilated caging (IVC) system, with a temperature of 21 ± 1 °C, 12 h light/dark cycle, 50–70% humidity, and ad libitum access to food (Chow or HFD) and water.

### 2.2. Generation of HFD-Induced Obesity Mouse Model

To induce obesity, only male mice were selected to avoid the influence of menstrual cycles of females. The obesity mouse models were generated with the supplement of HFD. Briefly, 4-week-old male mice with different genotypes were divided into two groups randomly. One group was fed with the basal diet (Dashuo, Chengdu, Sichuan, China), while the other was supplied with the high-fat diet (HFD, 45% fat, MD12032, Medicience, Yangzhou, China). Indicated diets were supplied for 14 weeks to induce obesity, and the bodyweight and food uptake of mice were measured weekly. After 14 weeks of indicated diet supplement, half of these mice were euthanized through cervical dislocation; the blood was collected by cardiac puncture and used for blood glucose analysis, and then the blood was centrifuged to obtain serum for blood biochemical assays detecting NEFA, TG, TC, HDL, LDL, leptin, and insulin levels. After that, the brain was dissected and the hypothalamus was collected in full and frozen in liquid nitrogen immediately for following qRT-PCR, Western blots, and biochemical assays; the epididymal, perirenal, inguinal white adipose tissue and brown adipose tissue were carefully collected for organ index measurement. The remaining half of the mice were perfused transcardially with phosphate buffer saline (PBS) and 4% paraformaldehyde (PFA) in order to isolate samples for following staining.

### 2.3. Glucose Tolerance Test (GTT) and Insulin Tolerance Test (ITT)

For the GTT experiment, overnight starved (20:00 p.m. to 8:00 a.m.) mice were injected with 0.75 g/kg glucose (Sigma, Darmstadt, Germany) by intraperitoneal injection at 8:00 a.m. Blood was collected from the tail vein at 0, 15, 30, 45, 60, 90, and 120 min after injection. The blood glucose concentration was measured using a rapid blood glucose meter (Roche, Foster City, CA, USA). For the ITT experiment, 6 h starved (8:00 a.m. to 14:00 p.m.) mice were injected with 1.5 U/kg insulin (Novo Nordisk, Bagsvaerd, Denmark) by intraperitoneal injection. Blood was collected from the tail vein at 0, 15, 30, 45, 60, 90, and 120 min after injection, and the blood glucose concentration was also measured using a rapid blood glucose meter. The data at all time points were statistically analyzed, and the mean ± standard deviation was plotted. The significance of differences between groups was analyzed using a two-way ANOVA method.

### 2.4. Quantitative Real-Time PCR

Hypothalamic tissue was obtained from the brain and the total RNA was extracted from samples using Animal Total RNA Isolation Kit (RE-03014, Foregene, Chengdu, China) according to manufacturer’s protocol. Then, ~1 μg total RNA was subjected to reverse transcription with RT EasyTM II kit (With gDNase) (RT-01023, Foregene, Chengdu, China) using the following conditions: 42 °C for 25 min and 85 °C for 5 min. After that, qRT–PCR was performed using Bio-Rad CFX96 Real-Time Detection System (Bio-Rad, Hercules, CA, USA) with Real-Time PCR EasyTM-SYBR Green I kit (QP-01014, Foregene, Chengdu, China) for three replicates, and the relative gene expression was normalized to internal control β-actin. Primer sequences for qRT–PCR are shown in Table 2.

### 2.5. Immunofluorescence Staining

Samples were fixed in 4% paraformaldehyde solution and embedded in paraffin. Sections of 5 μm length were mounted on slides using an Ultra-Thin Semiautomatic Microtome (S710, RWD Life Science Co., Ltd., Shenzhen, China). Then, the slides were deparaffinized, rehydrated, and underwent high-pressure antigen retrieval with citrate buffer (pH 6.0), before they were blocked with blocking buffer (1× PBS + 10% donkey serum + 0.01 g/mL BSA + 0.3% Triton X-100) at RT for 60 min. After blocking, the sections were incubated at 4 °C overnight with the primary antibodies, which were diluted in PBS with 1% donkey serum, 0.01 g/mL BSA, and 0.3% Triton X-100. After washing with PBS three times, the slides were incubated with the secondary antibodies in darkness at RT for 90 min. The antibodies used are listed in Table 3. After that, the slides were washed three times with 1× PBS, and the coverslips were mounted using ProLong Gold with DAPI mounting medium (P36962, Invitrogen, Eugene, OR, USA) for cell nuclei staining and photographed with a microscope (BX61VS, Olympus, Tokyo, Japan).

### 2.6. Western Blots

Brain samples were dissected and immediately frozen in liquid nitrogen. Total protein was extracted from tissues or cells by mincing them with sonication in sample lysis buffer (2% SDS with proteinase inhibitors and phosphatase inhibitor). The protein concentration was measured with BCA Protein Assay Kit (PC0020, Solarbio, Beijing, China). For Western blots, the sample containing 5–10 µg of protein was loaded into SDS-PAGE gels. Separated proteins on gels were electro-transferred to polyvinylidene difluoride membranes (PVDF, Millipore, Darmstadt, Germany) using the Trans-Blot^®^ Turbo™ transfer system (BioRad, Tokyo, Japan), followed by blocking with 5% skimmed milk in TBST for 1 h. Then, an overnight incubation of primary antibodies (Table 3) was performed. After washing with TBST three times, the secondary antibodies (1:10,000; Abclonal, Wuhan, China) were incubated for 1 h at room temperature, followed by another three washes with TBST. Finally, protein signals were detected and scanned using the Quantitative Fluorescence Imaging Systems (ChampChemi 910, Beijing SinSage Technology, Beijing, China).

### 2.7. Immunoprecipitation

For immunoprecipitation with mouse hypothalamus, protein was extracted from samples by mincing them with scissors on ice in immunoprecipitation (IP) buffer (50 mM Tris, 150 mM NaCl, 0.5% NP40, pH 7.5) containing protease inhibitor cocktail, and 1 mM PMSF. For immunoprecipitation with cultured PC12 cells to detect protein ubiquitinoylation, 24 h after the co-transfection of Myc-Ub and HA-PGC-1α plasmids, PA (100 μM) treatment was performed for 12 h, 24 h, and 36 h; then, the cell lysates were obtained by homogenizing cells in the same IP buffer mentioned above. Then, a centrifugation (14,000× *g* for 10 min, 4 °C) was performed to obtain the supernatant and the protein concentration was measured with BCA Protein Assay Kit (PC0020, Solarbio, Beijing, China) according to the manufacturer’s protocol. After the adjustment of protein concentration, every 200 μL of lysates were incubated overnight at 4 °C with 50 μL pretreated Protein A/G beads (B23202, Bimake, Beijing, China), which were first incubated with 5 mg/mL antibody for 1 h at 4 °C. The immunocomplex was washed three times with washing buffer (50 mM Tris, 150 mM NaCl, 0.5% detergent (NP40), pH 7.5) and resuspend using 1× loading buffer under 100 °C water bath heating for 10 mins. Finally, proteins were separated by SDS-PAGE and immunoblotted with indicated antibodies.

### 2.8. Cell Culture

PC12 and 293T cells were purchased from Cell Bank of the Chinese Academy of Sciences. The cells were validated with short tandem repeat analysis and maintained with DMEM containing 10% FBS (ST30-3302P, PAN) and 1% antibiotic/antimycotic (10,000 Units/mL penicillin and 10,000 μg/mL streptomycin) (Gibco, Grand Island, NY, USA) in an incubator under an atmosphere of 5% CO_2_ at 37 °C. To detect the induction of LanCL1 expression, PC12 cells were transfected with Prk5-HA-PGC-1α plasmids (4 μg per well of a six-well plate) using TransEasyTM transfection reagent (TEO-01012, Foregene, Chengdu, China) or treated with the indicated dose of PA (sigma, P5585-25G) for the indicated time. Finally, cells were harvested for qRT–PCR or Western blotting. For SP1 inhibition, 24 h after Prk5-HA-PGC-1α transfection, 20 nM Mithramycin A (dissolved in 0.1% DMSO) treatment was performed for another 24 h.

For detecting ROS production, fluorogenic dye 2,7-dichlorofluorescein diacetate (DCHF-DA, S0033M, Beyotime, Shanghai, China) was used. Cultured cells were treated with 100μM PA for 1 h, 2 h, or 4 h, and the DCFH-DA was diluted in serum-free medium at 1:1000 to a final concentration of 10 mmol/L. The cell culture medium was removed, the appropriate volume of diluted DCFH-DA was added, and the cells were incubated at 37 °C for 20 min. The cells were then washed three times with serum-free cell culture solution to fully remove redundant DCFH-DA. Finally, with nuclei labeled by Hoechst (C0003, Beyotime, Shanghai, China), the oxidized dyes in cells were visualized and photographed with a microscope (BX61VS, Olympus, Tokyo, Japan).

### 2.9. Detection of the Ratio of NADPH/NADP, GSH/GSSG, and MDA

Approximately 20 mg of each sample was used to detect NADPH/NADP and GSH/GSSG after homogenization with the indicated isolation buffer, and the indicated amount of sample was processed for detection using commercial kits according to the manufacturers’ instructions (K347, Biovision (Milpitas, CA, USA), for NADPH/NADP; S0053, Beyotime (Shanghai, China), for GSH/GSSG; K454, Biovision (Milpitas, CA, USA), for MDA). The absorbance of the samples was measured using a plate reader (Thermo, Waltham, MA, USA), and the values used for calculating the ratio were calculated using a standard curve.

### 2.10. Quantification and Statistical Analysis

Data represent the mean ± standard deviation (SD) or mean ± standard error of the mean (SEM). One-way ANOVAs or two-tailed Student’s *t*-test were performed for the statistical significance analysis using GraphPad Prism software (Version 9.0, San Diego, CA, USA): * *p* < 0.05, ** *p* < 0.01, *** *p* < 0.001, **** *p* < 0.0001.

## 3. Results

### 3.1. Neuronal Expression of Hypothalamic LanCL1 Corelates with HFD-Induced Obesity

**Figure 1:**
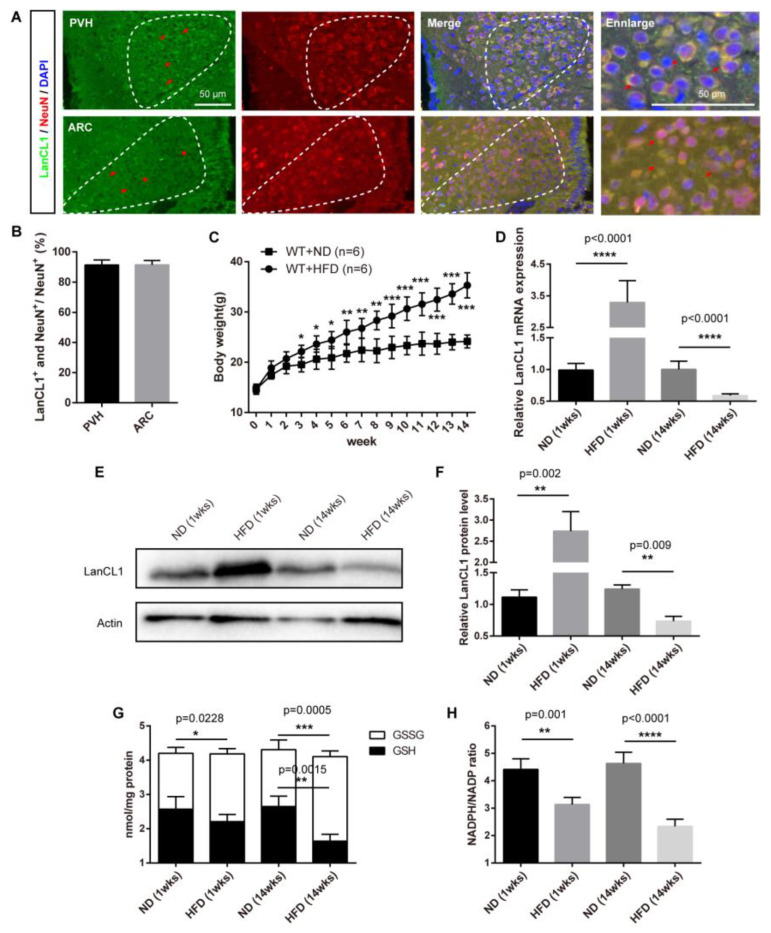
Neuronal expression of hypothalamic LanCL1 correlates with HFD-induced obesity. (**A**,**B**) Representative images and quantification showing the co-labeling of LanCL1 (green) with NeuN (red) in the PVH and ARC of hypothalamus in mice. Arrows indicate positive signals. (**C**) Bodyweight of mice supplied with normal died (ND) and high-fat diet (HFD). Statistical analysis was performed comparing between ND and HFD at each same time point. (**D**) qRT–PCR showing the fluctuant expression pattern of hypothalamic LanCL1 mRNA along with HFD exposure. Error bars indicate SD, n = 4. Statistical analysis was performed by two-tailed Student’s *t*-test. (**E**,**F**) Western blots and quantification showing the fluctuant expression pattern of hypothalamic LanCL1 protein along with HFD exposure. Error bars indicate SD, n = 3. Statistical analysis was performed by two-tailed Student’s *t*-test. (**G**,**H**) Decreased NADPH/NADP ratio and the levels of GSH and GSSS in the hypothalamus of mice exposed to HFD. Error bars indicate SEM, n = 4. Statistical analysis was performed by two-tailed Student’s *t*-test. * *p* < 0.05, ** *p* < 0.01, *** *p* < 0.001, **** *p* < 0.0001.

We previously showed the specifical expression of LanCL1 in neurons of mouse brain [20]. Consistently, in the hypothalamus, we found cytoplasmic concentrated LanCL1 co-labeled with NeuN (~98%), a neuron-specific nuclear protein (Figure 1A,B) [27]. Obesity affects hypothalamic redox balance and causes oxidative stress [28]. Interestingly, during the progress of HFD-induced obesity (Figure 1C), we noticed a fluctuant expression pattern of the antioxidant gene LanCL1 in the hypothalamus. Early before the onset of obesity, we found increased mRNA and protein levels of hypothalamic LanCL1 in mice with one-week supplement of HFD, while robust suppression of this was present in obese mice supplied with HFD for 14 weeks (Figure 1D–F). The attenuated hypothalamic LanCL1 indicated an overwhelmed antioxidant defense during obesity progress, as we found accumulating redox imbalance in the hypothalamus, showed by levels of increased oxidized glutathione and decreased reduced glutathione, as well as a reduced ratio of NADPH and NADP (Figure 1G,H). These data suggested potential important roles of hypothalamic LanCL1 in regulating energy metabolism and obesity progress.

**Figure 2:**
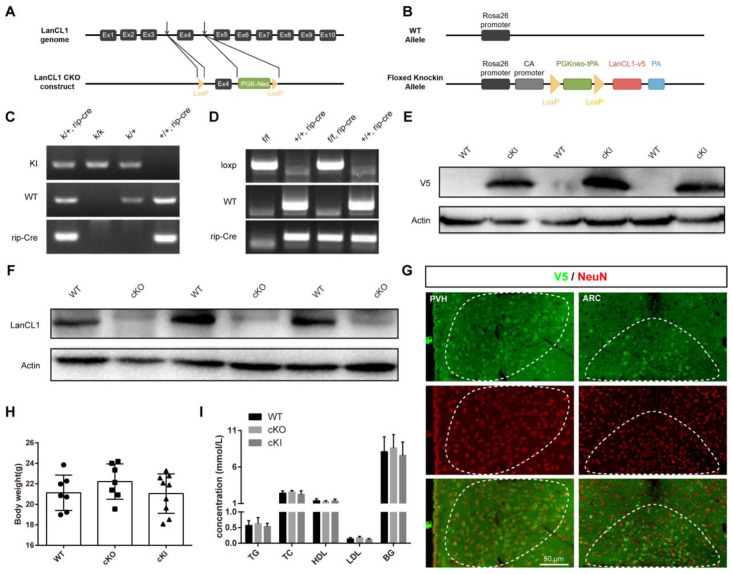
Generation of conditional LanCL1 knockout (cKO) and knockin (cKI) mice. (**A**,**B**) Overview of the targeting strategy for generating LanCL1 knockout and knockin mice. (**C**,**D**) Genotyping PCR validating LanCL1 cKI and cKO mice. (**E**,**F**) The hypothalamic overexpression of LanCL1 protein (**C**) and absence of it (**D**) are validated by Western blots. (**G**) Representative images of hypothalamic PVH and ARC area showing immunofluorescence labeling of V5 (green) and NeuN (red) in LanCL1 cKI WT mice. (**H**) Bodyweight of LanCL1 WT (n = 7), cKO (n = 7), and cKI (n = 9) mice at 8 weeks of age. Error bars indicate SD, n = 4. F = 0.9657 and DF = 22 according to one-way ANOVAs that analyze the statistical difference compared with WT group. (**I**) Quantification showing the serum metabolic parameters in LanCL1 WT, cKO, and cKI mice at 8 weeks of age. TG, triglycerides (F = 0.3243, DF = 13); TC, total cholesterol (F = 0.9247, DF = 13); LDL, low-density lipoprotein (F = 2.149, DF = 13); HDL, high-density lipoprotein (F = 0.4991, DF = 13); BG, blood glucose (F = 0.277, DF = 131). Error bars indicate SEM, n = 4. Statistical analysis was performed by one-way ANOVAs that analyze the statistical difference compared with WT group.

To further elucidate this, we generated two conditional transgenic mouse models; with *LanCL1*^flox/flox^ mice and *Rosa26*-*LanCL1*-V5 transgenic mice we generated before [20,21] (Figure 2A,B), we crossed them with rip-Cre mice that express Cre in hypothalamic cells and pancreatic β cells [17] to specifically delete or overexpress LanCL1 in the hypothalamus (named cKO and cKI mice hereinafter). After the genotype validation (Figure 2C,D), we confirmed the deficiency and overexpression of LanCL1 in the hypothalamus with Western blots and fluorescent immunostaining, and we noticed that rip-Cre-mediated LanCL1 overexpression was concentrated in hypothalamic neurons (Figure 2E–G). At the adult age, both cKO and cKI mice had comparable bodyweight to WT mice (Figure 2H), and LanCL1 knockout or knockin did not alter overall body metabolism of mice, according to the blood biochemical examination results (Figure 2I).

### 3.2. Loss of Hypothalamic LanCL1 Aggravates HFD-Induced Obesity

**Figure 3:**
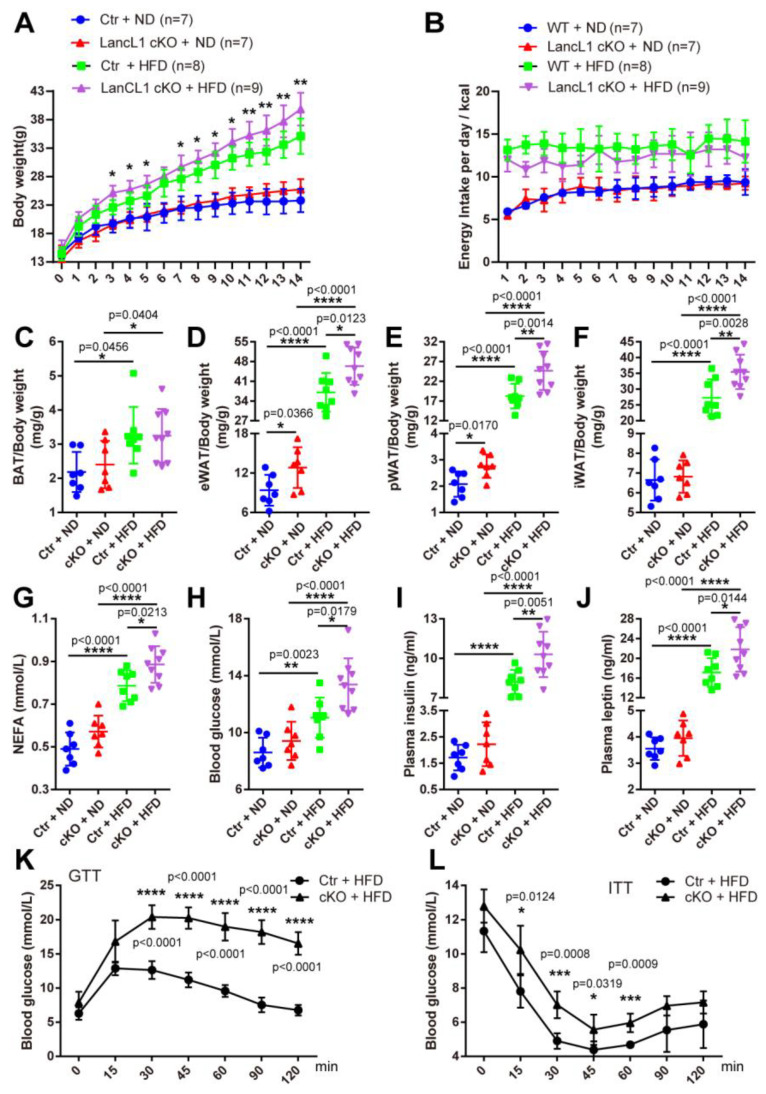
Loss of hypothalamic LanCL1 aggravates HFD-induced obesity. (**A**) Bodyweight for control (Ctr, LanCL1f/f or LanCL1f/+) and LanCL1 cKO mice during consecutive feeding of ND or HFD for 14 weeks. Statistical analysis was performed to compare between Ctr + HFD and cKO + HFD at each same time point. (**B**) Daily energy intake for Ctr and LanCL1 cKO mice during ND and HFD feeding. (**C**–**F**) Organ index of BAT ((**C**), F = 4.485, DF = 30), eWAT ((**D**), F = 90.57, DF = 30), pWAT ((**E**), F = 103.2, DF = 30) and iWAT ((**F**), F = 93.84, DF = 30) of LanCL1 Ctr and cKO mice supplied with ND and HFD. Error bars indicate SD. Statistical analysis was performed by one-way ANOVAs. (**G**–**J**) Plasma NEFA ((**G**), F = 43.43, DF = 30), blood glucose ((**H**), F = 16.61, DF = 30), plasma insulin ((**I**), F = 111.2, DF = 30), and plasma leptin ((**J**), F = 81.29, DF = 30) levels of LanCL1 Ctr and cKO mice supplied with ND and HFD. Error bars indicate SEM. Statistical analysis was performed by one-way ANOVAs. (**K**,**L**) Blood glucose levels during GTT (**K**) and ITT (**L**) in LanCL1 Ctr and cKO mice after 14 weeks of HFD exposure. Error bars indicate SEM, n = 5. Statistical analysis was performed to compare between Ctr + HFD and cKO + HFD at each same time point. * *p* < 0.05, ** *p* < 0.01, *** *p* < 0.001, **** *p* < 0.0001.

Considering the maintenance of hypothalamic redox balance implicated in systemic metabolic regulation [17,29,30], we wondered if disturbed antioxidant defense mediated by LanCL1 would affect the progress of obesity and its related metabolic disorders. Thus, we first generated HFD-induced obese mouse models with LanCL1 cKO male mice. After 14 weeks of consumption of HFD, cKO mice gained more bodyweight than the control mice, while no differences in bodyweight gain were observed between them and those supplied with a normal diet, and the difference in bodyweight gain did not result from energy uptake disparity (Figure 3A,B). Consistent with this finding, we found excessive accumulation of epididymal/perirenal/inguinal white adipose tissue, but not brown adipose tissue, in the obese mice, which was accumulated more in the cKO mice than in the Ctr mice (Figure 3C–F). Also, an increased level of blood non-esterified fatty acids (NEFAs) was observed in the cKO obese mice, compared with the Ctr obese ones (Figure 3G). In addition to these, we noticed differences in the impairment of glucose homeostasis induced by obesity between LanCL1 Ctr and cKO mice, and the obese cKO mice had higher levels of blood glucose, insulin, and leptin than the obese Ctr mice (Figure 3H–J). To further evaluate the glucose metabolism, a glucose tolerance test (GTT) was first performed on LanCL1 Ctr and cKO mice after HFD feeding, showing much higher blood glucose level in obese cKO mice (Figure 3K). Then, with an insulin tolerance test (ITT), we also found increased blood glucose level in obese cKO mice (Figure 3L). Collectively, these data indicated an essential role of hypothalamic LanCL1 to maintain the glucose homeostasis and mitigate obesity progress induced by HFD.

### 3.3. Hypothalamic Transgene of LanCL1 Attenuates HFD-Induced Obesity 

**Figure 4:**
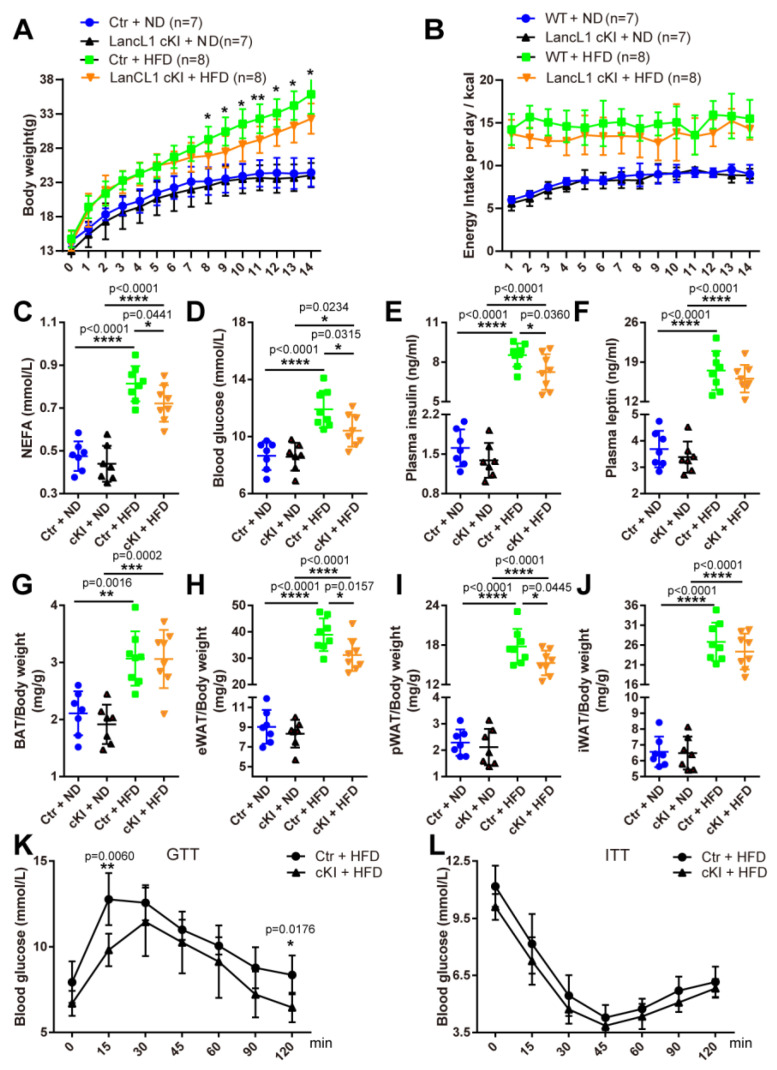
Hypothalamic transgene of LanCL1 attenuates HFD-induced obesity. (**A**) Bodyweight for Ctr (LanCL1k/k or LanCL1k/+) and LanCL1 cKI mice during consecutive feeding of ND or HFD for 14 weeks. Error bars indicate SD. Statistical analysis was performed to compare between Ctr + HFD and cKO + HFD at each same time point. (**B**) Daily energy intake for Ctr and LanCL1 cKI mice during ND and HFD feeding. Error bars indicate SD. (**C**–**F**) Plasma NEFA ((**C**), F = 38.86, DF = 29), blood glucose ((**D**), F = 15.34, DF = 29), plasma insulin ((**E**), F = 138.8, DF = 29), and plasma leptin ((**F**), F = 88.33, DF = 29) levels of LanCL1 Ctr and cKI mice supplied with ND and HFD. Error bars indicate SEM. Statistical analysis was performed by one-way ANOVAs. (**G**–**J**) Organ index of BAT ((**G**), F = 14.53, DF = 29), eWAT ((**H**), F = 85.19, DF = 29), pWAT ((**I**)**,** F = 173.1, DF = 29), and iWAT ((**J**), F = 74.25, DF = 29) of LanCL1 Ctr and cKI mice supplied with ND and HFD. Error bars indicate SD. Statistical analysis was performed by one-way ANOVAs. (**K**,**L**) Blood glucose levels during GTT (**K**) and ITT (**L**) in LanCL1 Ctr and cKI mice after 14 weeks of HFD exposure. Error bars indicate SEM, n = 5. Statistical analysis was performed to compare between Ctr + HFD and cKO + HFD at each same time point * *p* < 0.05, ** *p* < 0.01, *** *p* < 0.001, **** *p* < 0.0001.

To further demonstrate the important roles of hypothalamic LanCL1 in mitigating obesity progress, obese mouse models with LanCL1 cKI mice were further generated. Compared with the Ctr mice, LanCL1 cKI mice had comparable metabolic parameters when supplied with normal diets (Figure 4C–F). In contrast, after they were exposed to HFD for 14 weeks, cKI mice gained less bodyweight with comparable energy intake (Figure 4A,B). Improvement in HFD-induced bodyweight gain was accompanied with less accumulation of white adipose tissue (eWAT, pWAT, iWAT), but not BAT (Figure 4G–J), as well as reduced blood NEFA level (Figure 4C) in obese cKI mice when compared with obese Ctr ones. As was expected, we also detected remarkably lower levels of blood glucose and insulin in obese cKI mice than in obese Ctr mice (Figure 4D,E). Furthermore, a reduced, but not significantly reduced, level of blood leptin was also observed in obese cKI mice (Figure 4F). To further evaluate the effects of hypothalamic LanCL1 overexpression on glucose metabolism, the GTT and ITT were also performed. While we observed a reduced trend of blood glucose at each time point in both tests, there were rarely significant differences (Figure 4K,L). All these results suggested that hypothalamic LanCL1 overexpression is protective, but not completely resistant, against HFD-induced obesity and disorders in metabolic homeostasis. 

### 3.4. Hypothalamic Inflammation Is Implicated in LanCL1-Correlated Protection against HFD-Induced Obesity 

**Figure 5:**
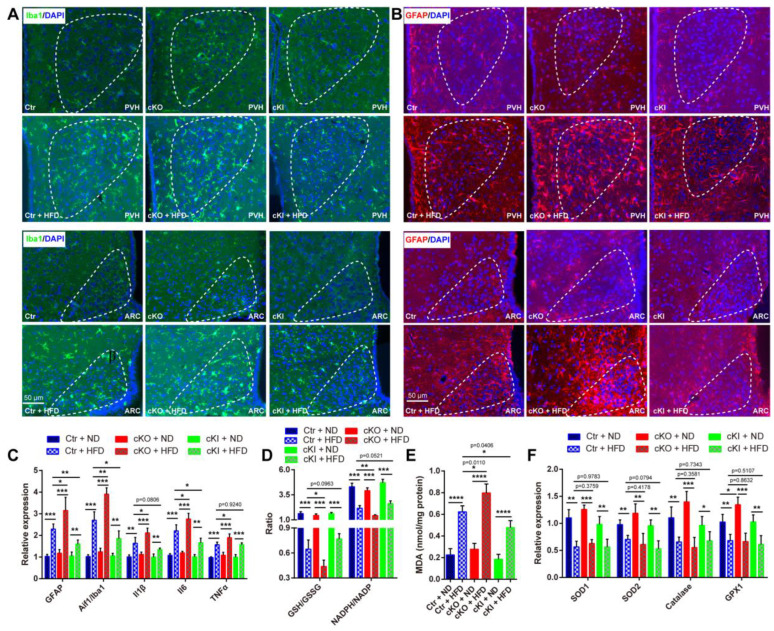
Hypothalamic inflammation is implicated in LanCL1-correlated protection against HFD-induced obesity. (**A**,**B**) Representative images of immunofluorescence staining for Iba1 (green) and GFAP (red) in PVH (**upper**) and ARC (**lower**) of hypothalamus of LanCL1 Ctr, cKO, and cKI mice supplied with ND or HFD for 14 weeks. (**C**) qRT-PCR showing relative mRNA abundance for Iba1 (F = 76.90, DF = 23), GFAP (F = 32.84, DF = 23) and proinflammatory cytokines (Il1β (F = 27.73, DF = 23), Il6 (F = 55.78, DF = 23), TNF-α (F = 40.41, DF = 23)) in hypothalamus of LanCL1 Ctr, cKO, and cKI mice fed with ND or HFD for 14 weeks. Error bars indicate SEM, n = 4. Statistical analysis was performed by one-way ANOVAs. (**D**) Quantification showing the GSH/GSSG (F = 71.70, DF = 23) and NADPH/NADP (F = 84.19, DF = 23) ratios in the hypothalamus of LanCL1 Ctr, cKO, and cKI mice supplied with ND or HFD for 14 weeks. Error bars indicate SEM, n = 4. Statistical analysis was performed by one-way ANOVAs. (**E**) MDA assay detecting lipid peroxidation products in hypothalamus of LanCL1 Ctr, cKO, and cKI mice supplied with ND or HFD for 14 weeks. Error bars indicate SEM, n = 4. F = 64.50 and DF = 23 according to one-way ANOVAs. (**F**) qRT-PCR showing relative mRNA abundance for SOD1 (F = 25.42, DF = 23), SOD2 (F = 12.98, DF = 23), Catalase (F = 14.80, DF = 23), and GPX1 (F = 16.52, DF = 23) in hypothalamus of LanCL1 Ctr, cKO, and cKI mice supplied with ND or HFD for 14 weeks. Error bars indicate SEM, n = 4. Statistical analysis was performed by one-way ANOVAs * *p* < 0.05, ** *p* < 0.01, *** *p* < 0.001, **** *p* < 0.0001.

Previous studies have reported the close relationship between oxidative stress and neuroinflammation. ROS is shown to be involved in regulating neuroinflammatory pathways such as IKKβ/NF-κB, and oxidative stress may play an important role in the induction of neuroinflammation; however, during the occurrence of neuroinflammation, activated microglia and astrocytes will produce more ROS through enzymes such as NOX2 or iNOS, which exacerbates the damage of nucleic acids, proteins, and lipids caused by oxidative stress, and induces apoptosis [31,32,33]. According to above results, we wondered if hypothalamic oxidative stress played an important role in HFD-induced hypothalamic inflammation and subsequent obesity progress. To address this, we first labeled the activated microglia and astrocytes, key regulators of inflammatory responses in the central nervous system, with specific markers Iba1 and GFAP, respectively. Consistent with previous studies, HFD exposure could promote the activation of microglia and astrocytes in the hypothalamus (Figure 5A,B). However, we noticed more Iba1^+^ and GFAP^+^ signals in LanCL1 cKO hypothalamus (1.5-fold for Iba1 and 2.1-fold for GFAP-positive cells per field compared with Ctr + HFD group, respectively), while fewer signals were detected in LanCL1 cKI hypothalamus (47% and 69% reduced positive cells per field for Iba1 and GFAP, respectively, compared with Ctr + HFD group) (Figure 5A,B). Also, this finding was validated by qRT-PCR detecting the expressions of hypothalamic Iba1 and GFAP (Figure 5C). To confirm this, expressions of hypothalamic proinflammatory cytokines (IL-1β, IL-6, TNF-α) were further evaluated, showing more aggravated inflammation in the obese LanCL1 cKO mice than in the obese Ctr ones, and attenuated inflammation was present in obese LanCL1 cKI mice (Figure 5C). As LanCL1 was widely proven to be an antioxidant gene, we then studied whether hypothalamic redox homeostasis was involved in LanCL1-mediated protection against HFD-induced neuroinflammation. With quantifications of the ratios of NADPH/NADP and GSH/GSSG, we observed more remarkable hypothalamic redox imbalance in obese LanCL1 cKO, while relatively more balanced redox homeostasis was present in obese LanCL1 cKI mice than in obese Ctr mice (Figure 5D). This finding was further validated by detecting lipid peroxidation products in the hypothalamus (Figure 5E). Differences in hypothalamic redox homeostasis and damages among these obese mice should be mediated by different expressions of LanCL1, as the expression of most of the common antioxidant genes was found to be suppressed in the hypothalamus of LanCL1 Ctr, cKO, and cKI mice supplied with HFD (Figure 5F). Taken together, these data revealed important roles of LanCL1-linked redox homeostasis in regulating HFD-induced hypothalamic inflammation.

### 3.5. PGC-1α–SP1–LanCL1 Axis Is Implicated in Hypothalamic Response to HFD Exposure

**Figure 6:**
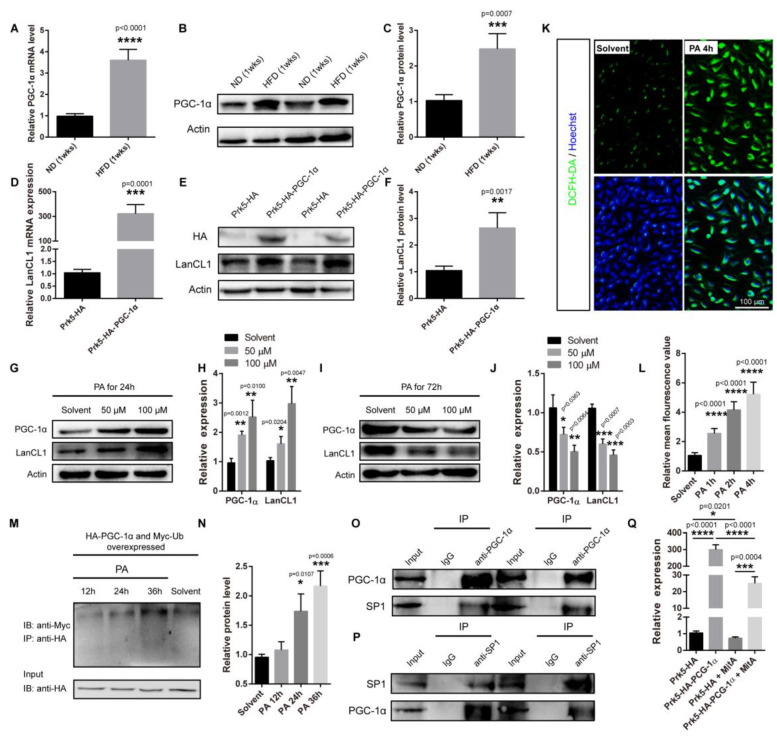
PGC-1α–SP1–LanCL1 axis is implicated in hypothalamic response to HFD exposure. (**A**) qRT–PCR showing increased hypothalamic PGC-1α expression in mice supplied with HFD for 1 week. Error bars indicate SEM, n = 4. Statistical analysis was performed by two-tailed Student’s *t*-test. (**B**,**C**) Western blots and quantification showing increased protein level of PGC-1α in hypothalamus of mice supplied with HFD for 1 week. Error bars indicate SEM, n = 4. Statistical analysis was performed by two-tailed Student’s *t*-test. (**D**) qRT–PCR showing increased LanCL1 expression in PC12 cells transfected with Prk5-HA-PGC-1α. Error bars indicate SEM, n = 4. Statistical analysis was performed by two-tailed Student’s *t*-test. (**E**,**F**) Western blots and quantification showing increased protein level of LanCL1 in PGC-1α-overexpressed PC12 cells. Error bars indicate SD, n = 4. Statistical analysis was performed by two-tailed Student’s *t*-test. (**G**–**J**) Western blots and quantifications showing protein levels of PGC-1α in PC12 cells with short-time (24 h, (**G**,**H**)) and long-time (72 h, (**I**,**J**)) treatment of palmitic acid (PA). Error bars indicate SD, n = 3. Statistical analysis was performed by two-tailed Student’s *t*-test and compared with solvent group (**K**,**L**) Representative images and quantification showing increased ROS production induced by short-time PA treatment, as indicated by DCFH-DA labeling. Error bars indicate SEM, n = 4. F = 121.5 and DF = 39 according to one-way ANOVAs. Statistical analysis was performed to compare with solvent group. (**M**,**N**) Western blots and quantification showing increased ubiquitinoylation of PGC-1α along with PA (100 μM) treatment. Error bars indicate SD, n = 3. F = 22.32 and DF = 11 according to one-way ANOVAs. Statistical analysis was performed to compare with solvent group. (**O**,**P**) Immunoprecipitation analysis performed with anti-PGC-1α antibody (**O**) or anti-SP1 antibody (**P**) in mouse hypothalamic samples displaying the interaction of PGC-1α with SP1. (**Q**) qRT–PCR showing suppressed expression of LanCL1 in PGC-1α-overexpressed cells when treated with SP1 inhibitor mithramycin A (MitA). Error bars indicate SEM, n = 3. F = 291.2 and DF = 11 according to one-way ANOVAs. Statistical analysis was performed to compare with Prk5-HA group. * *p* < 0.05, ** *p* < 0.01, *** *p* < 0.001, **** *p* < 0.0001.

As a center integrating metabolic feedback and regulating energy homeostasis, hypothalamic function is affected by lots of signals. The peroxisome proliferator activated receptor gamma coactivator 1 α (PGC-1α) is a transcriptional co-activator involved in regulating lots of metabolic and energy pathways, as well as cellular antioxidant defense [34]. We and other researchers reported that the hypothalamic expression of PGC-1α would be suppressed by a long-time exposure to HFD, and in response to blood long-chain fatty acid palmitic acid (PA) in obese subjects, hypothalamic PGC-1α may function to protect against HFD exposure [35,36]. In this study, we further found an increased expression of hypothalamic PGC-1α in mice supplied with HFD at the early stage (Figure 6A–C), and the same fluctuated expression patterns of PGC-1α and LanCL1 in the hypothalamus in response to HFD exposure (Figure 1D) made us wonder whether a direct correlation was present between them. To address this, we overexpressed PGC-1α in culture cells and found that PGC-1α could promote a robust expression of both LanCL1 mRNA and protein (Figure 6D–F). Then, to imitate obese status in vitro, we subjected PC12 cells to PA, and observed that short-time PA treatment promoted the expressions of PGC-1α and LanCL1 (Figure 6G,H), while long-time PA treatment suppressed their expressions (Figure 6I,J). Furthermore, we found that increased expression of PGC-1α may be a consequence of accumulated ROS production induced by PA treatment (Figure 6K,L), as PGC-1α was widely demonstrated to be ROS-inducible [37,38]. In contrast, for reduced expression of PGC-1α under longtime PA treatment, we thought this resulted from its increased degradation, as we noticed that long-time PA treatment promoted the ubiquitinoylation of PGC-1α (Figure 6M,N). These data reveal the involvement of PGC-1α in regulating LanCL1 expression in response to HFD exposure. However, as a transcriptional co-activator, PGC-1α is not supposed to transcriptionally regulate LanCL1 directly. We previously showed that transcriptional factor SP1 mediated the transcriptional regulation of LanCL1 in response to ROS. Thus, we wondered if PGC-1α participated in the regulation of the SP1–LanCL1 axis. Through immunoprecipitation assay performed with a PGC-1α antibody using mouse hypothalamus lysates, we found that PGC-1α interacted with SP1, which was validated by a further immunoprecipitation assay immunoprecipitated with anti-SP1 antibody (Figure 6O,P). In addition, we noticed that inhibited-activity SP1 with its inhibitor mithramycin A significantly suppressed the inducibility of LanCL1 in response to PGC-1α overexpression. All these results provided evidence showing PGC-1α was involved in the response of LanCL1 to HFD exposure, and suggested an important role of the PGC-1α–SP1–LanCL1 axis in protecting against HFD-induced obesity.

## 4. Discussion

Hypothalamic inflammation and oxidative stress/damages are well-documented in obesity, especially diet-induced obesity. As an early event upon HFD exposure [35], hypothalamic inflammation is implicated in the development and progress of obesity, playing a role not only as an important driver of impaired energy homeostasis, but also a critical contributor to obesity-induced comorbidities [9,39,40]. Multiple key inflammatory responders, such as JNK and IκB kinase, are involved in activating hypothalamic inflammation in response to a high-fat diet and the intervention of inflammatory processes in the hypothalamus that ameliorate the progress of obesity [12,13,39]. Similar to inflammation, chronic oxidative stress is thought to be a contributor to the pathophysiological conditions of obesity and its comorbidities, such as hypertension, atherosclerosis, metabolic syndrome, and type 2 diabetes [41,42,43,44]. Under normal conditions, hypothalamic reactive oxygen species (ROS) function to maintain energy homeostasis through balancing the activation between POMC and NPY/AgRP neurons, but excessive ROS would damage these hypothalamic neurons, resulting in disturbed energy homeostasis and obesity [29,45,46]. With HFD consumption, elevated expression of NADPH oxidase, which is a major source of ROS, is present in the hypothalamus, while ablation of hypothalamic p22^phox^, the activator of NADPH oxidase, is protective against HFD-induced obesity [47]. To support the implications of hypothalamic redox homeostasis in HFD-induced obesity, remarkably increased oxidative damages and decreased antioxidant capacity were found in the hypothalamus of experimental animals supplied with HFD [28,48,49]. While these and other studies have focused on hypothalamic inflammation and oxidative stress in the development and progress of obesity, the evidence describing the correlations between these two pathophysiological conditions is not enough. Here, in this study, with genetic mouse models targeting antioxidant gene LanCL1 in the hypothalamus, we evaluated the effects of altered hypothalamic antioxidant defense on HFD-induced obesity. We found elevated hypothalamic LanCL1 expression in response to HFD exposure at the early stage, while a suppressed level was observed after long-time HFD feeding. This fluctuant expression pattern of hypothalamic LanCL1 displayed the progress of overwhelmed LanCL1-mediated antioxidant defense along with HFD exposure and suggested the importance of hypothalamic LanCL1 in regulating energy homeostasis. In support of this, hypothalamus-specific deletion of LanCL1 aggravated the HFD-induced progress of obesity and the impairment of glucose metabolism, while hypothalamus-specific overexpression of LanCL1 improved these defects even with remarkably decreased expressions of some other common antioxidant enzymes. Although the LanCL1 transgene is not completely against all the defects or phenotypes induced by HFD, it should be a consequence of overwhelmed LanCL1-mediated defense caused by long-time HFD exposure. Furthermore, perturbed expression of hypothalamic LanCL1 affected the hypothalamic inflammatory response under HFD exposure, suggesting critical roles of redox homeostasis in regulating inflammation in the hypothalamus during the development and progress of obesity.

PGC-1α is a transcriptional coactivator that interacts with a broad range of transcription factors and is implicated in the regulation of mitochondrial biogenesis, cellular respiration, energy metabolism, cellular antioxidant defense, etc. [50,51]. Enriched in tissues with high energy demands, PGC-1α is involved in the pathogenesis of obesity and its comorbidities, and perturbed PGC-1α expression is found in lots of tissues, like adipose tissue, liver, skeletal muscle, and brain, both in human obese subjects and HFD-induced obese animal models [36,52,53,54,55,56,57,58]. For roles of hypothalamic PGC-1α activity in the development and progress of obesity, contradictory results were reported. Ma et al. revealed that neuronal inactivation of PGC-1α was protective against HFD-induced obesity [59], and this was consistent with the results shown in PGC-1α whole-body knockout mice [60]. However, Morselli et al. demonstrated that reduced hypothalamic PGC-1α, targeting estrogens and estrogen receptor α (ERα), contributed to hypothalamic inflammation and obesity progress in response to HFD-induced palmitic acid (PA) accumulation in the brain in a sex-specific manner [35]. Previously, we also found suppressed hypothalamic PGC-1α upon long-time HFD exposure [36]. Here, in this study, we further showed a fluctuated expression pattern of hypothalamic PGC-1α, just like that of LanCL1, along with HFD exposure in vivo and upon PA treatment in vitro. We hypothesized that this phenotype represented the process of hypothalamic antioxidant defense regulated by PGC-1α being overwhelmed by HFD exposure, which contributed to obesity, and LanCL1 may be a target of PGC-1α. In support, we found that overexpression of PGC-1α promoted the expression of LanCL1, and this regulation activity was mediated by an interaction of PGC-1α with SP1, a zinc finger transcription factor directly binding to the LanCL1 promoter [22]. We also showed that the elevated PGC-1α and LanCL1 expressions at the early stage of HFD/PA treatment should be mediated by increased ROS production, while suppressed PGC-1α and LanCL1 expressions after long-time HFD/PA treatment were a consequence of increased ubiquitin-mediated PGC-1α degradation. Our findings provided direct evidence showing the implications of the PGC-1α–SP1–LanCL1 axis in the development and progress of HFD-induced obesity. However, limitations are present in this work, as it only was conducted with males. Hypothalamic PGC-1α is implicated in different responses in male and female mice supplied with HFD. Therefore, it is interesting but unknown whether a sexual dimorphic effect is present in the hypothalamic PGC-1α–SP1–LanCL1 axis in regulating the development and progress of obesity.

## 5. Conclusions

In summary, neuroinflammation and oxidative stress in the hypothalamus are widely reported to be implicated in the process of HFD-induced obesity, and our study provides a new perspective showing that the manipulation of hypothalamic antioxidant defense would affect HFD-induced neuroinflammation, as well as the development and progress of obesity. Also, our work demonstrates that the PGC-1α-coordinated activity of the SP1–LanCL1 axis couples HFD exposure with obesity development/progress. 

## Figures and Tables

**Table 1:** Primers for genotyping PCR.

Gene Type	Primer	Sequence
WT	F	5′-CGA ATC GTG TCA TCA TCT GG-3′
	R	5′-TGC ACT AAA AAT GCC GTC TG-3′
*FloxP*	F	5′-GCC CAA TTC CGA TCA TAT TC-3′
	R	5′-CTT AGC CGA GGC AGA AAC AC-3′
*Rosa-KI*	F	5′-AAA GTC GCT CTG AGT TGT TAT-3′
	R	5′-GGG CGT ACT TGG CAT ATG AT-3′
*rip-Cre*	F	5′-ACT CCA AGT GGA GGC TGA GA-3′
	R	5′-TCC TTC CAC AAA CCC ATA GC-3′

**Table 2:** Primers for qRT–PCR.

Gene	Primer	Sequence
*β-Actin* (*mouse*)	F	5′-AGA GGG AAA TCG TGC GTG AC-3′
	R	5′-CAA TAG TGA TGA CCT GGC CGT-3′
*PGC-1α*	F	5′-TAT GGA GTG ACA TAG AGT GTG CT-3′
	R	5′-CCA CTT CAA TCC ACC CAG AAA G-3′
*IL-6*	F	5′-CTT CCA TCC AGT TGC CTT CTT G-3′
	R	5′-AAT TAA GCC TCC GAC TTG TGA AG-3′
*IL-1β*	F	5′-CCC CAG GGC ATG TTA AGG AG-3′
	R	5′-TCT TGG CCG AGG ACT AAG GA-3′
*TNF-α*	F	5′-ACG GCA TGG ATC TCA AAG AC-3′
	R	5′-GTG GGT GAG GAG CAC GTA G-3′
*GFAP*	F	5′-CAA CGT TAA GCT AGC CCT GGA CAT-3′
	R	5′-CTC ACC ATC CCG CAT CTC CAC AGT-3′
*Iba1*	F	5′-CTT TTG GAC TGC TGA AGG C-3′
	R	5′-CAA CGT TAA GCT AGC CCT GGA CAT-3′
*LanCL1*	F	5′-CCT TCA GGT GAA CCA AGG AA-3′
	R	5′-AGA TCA CGT CAG CAC ACT GC-3′
*SOD1*	F	5′-AAC CAG TTG TGT TGT CAG GAC-3′
	R	5′-CCA CCA TGT TTC TTA GAG TGA GG-3′
*SOD2*	F	5′-TGG ACA AAC CTG AGC CCT AAG-3′
	R	5′-CCC AAA GTC ACG CTT GAT AGC-3′
*Catalase*	F	5′-TGG CAC ACT TTG ACA GAG AGC-3′
	R	5′-CCT TTG CCT TGG AGT ATC TGG-3′
*GPX1*	F	5′-TAC ACC GAG ATG AAC GAT CTG-3′
	R	5′-ATT CTT GCC ATT CTC CTG GT-3′

**Table 3:** Antibodies used in this study.

Target Protein	Producer	Product Code	Application
NeuN	CST (Danvers, MA, USA)	24307	IF (1:200)
V5-Tag	CST (Danvers, MA, USA)	13202S	IF (1:100) WB (1:1000)
GFAP	Sigma-Aldrich (Darmstadt, Germany)	MAB360	IF (1:1000)
Iba1	Abcam (Waltham, MA, USA)	ab178847	IF (1:1000)
aRab-488 Alexa Fluor	Jackson ImmuneResearch (West Grove, PA, USA)	711-547-003	IF (1:50)
aRab-594 Alexa Fluor	Invitrogen (Eugene, OR, USA)	A-11037	IF (1:500)
aGoat-488 Alexa Fluor	Invitrogen (Eugene, OR, USA)	A-11055	IF (1:1000)
aM-594 Alexa Fluor	Invitrogen (Eugene, OR, USA)	A-21203	IF (1:1000)
β-Actin Rabbit mAb	Abclonal (Wuhan, China)	AC026	WB (1:100,000)
Myc-Tag	Sigma-Aldrich (Darmstadt, Germany)	05-419	IP (1:50) WB (1:1000)
HA-Tag	ABclonal (Wuhan, China)	AE008	IP (1:50) WB (1:1000)
SP1	Santa Cruz Biotechnology (Dallas, TX, USA)	sc-17824	IP (1:50) WB (1:1000)
PGC-1α	Santa Cruz Biotechnology (Dallas, TX, USA)	sc-518025	IP (1:50) WB (1:500)
Ubiquitin	Santa Cruz Biotechnology (Dallas, TX, USA)	sc-8017	WB (1:1000)
HRP Goat Anti-Mouse IgG (H + L)	ABclonal (Wuhan, China)	AS003	WB (1:10,000)
HRP Goat Anti-Rabbit IgG (H + L)	ABclonal (Wuhan, China)	AS014	WB (1:10,000)

## Data Availability

The original data of the current study are available from the corresponding authors upon reasonable request.
